# Clear cell endometrial carcinoma precursors: presentation of two cases and diagnostic issues

**DOI:** 10.1186/s13000-021-01154-8

**Published:** 2021-10-25

**Authors:** Angela Santoro, Antonio Travaglino, Frediano Inzani, Damiano Arciuolo, Giuseppe Angelico, Nicoletta D’Alessandris, Giulia Scaglione, Michele Valente, Maurizio Martini, Antonio Raffone, Gian Franco Zannoni

**Affiliations:** 1 Unità di Ginecopatologia e Patologia Mammaria, Dipartimento Scienze della Salute della Donna, del Bambino e di Sanità Pubblica, Fondazione Policlinico Universitario A. Gemelli IRCCS, Largo A. Gemelli 8, 00168 Roma, Italy; 2 Department of Advanced Biomedical Sciences, Pathology Section, School of Medicine, University of Naples “Federico II”, Via Sergio Pansini, 5, 80131 Naples, Italy; 3 Division of Pathology, Policlinico Gemelli Foundation, Catholic University of the Sacred Heart, Rome, Italy; 4 Gynecology and Obstetrics Unit, Department of Neuroscience, Reproductive Sciences and Dentistry, School of Medicine, University of Naples Federico II, Naples, Italy; 5 Istituto di Anatomia Patologica, Università Cattolica del Sacro Cuore, Largo A. Gemelli 8, 00168 Roma, Italy

**Keywords:** Clear cell endometrial carcinoma, Serous endometrial carcinoma, Endometrial intraepithelial carcinoma, Immunohistochemistry, Case report

## Abstract

**Background:**

The precursors of clear cell endometrial carcinoma (CC-EC) are still undefined. Here, we deal with the diagnostic issues related to CC-EC precursors by presenting a morphological, immunophenotypical and molecular study of two representative cases and discussing the relevant literature.

**Case presentation:**

Our and previous cases suggest that clear cell endometrial intraepithelial carcinoma (CC-EIC) is a real entity, which may be distinguished from metaplastic/reactive changes and from its serous counterpart. CC-EIC appears associated with atrophic polyps and may be diagnosed based on morphological and immunophenotypical features of CC-EC in the absence of invasive disease. We described a p53-mutant putative precursor characterized by high-grade nuclei in the absence of other distinctive features. Two putative low-grade precursors resembled atypical tubal metaplasia and endometrial intraepithelial neoplasia, although immunohistochemistry could not support their relationship with CC-EC.

**Conclusions:**

In conclusion, pathologists should be aware of the existence of CC-EIC, since its correct diagnosis may be crucial for a correct patient management. Although several putative earlier precursors have been described, they does not show univocal features that allow their recognition in the common practice. Further studies are necessary in this field.

## Background

CC-EC is an uncommon neoplasm which has typically been regarded as a “type II” endometrial carcinoma, since it arises in atrophic endometrium, shows aggressive behavior and is not estrogen-related [1, 2]. Despite not having specific recognized mutations, CC-EC shows peculiar morphological and immunophenotypical features that allow its distinction from endometrioid and serous carcinoma. Histologically, CC-EC shows a combination of papillary, tubulocystic and solid architecture; tumor cells are cuboidal/polygonal, show cytoplasmic clearing/eosinophilia and a typical “hobnail” appearance, in the absence of overt stratification. Nuclear pleomorphism and mitotic activity are often not striking [2, 3]. By immunohistochemistry, CC-EC is characterized by negativity for estrogen receptor (ER), progesterone receptor (PR) and WT1, and positivity for Napsin A, HNF1β (hepatocyte nuclear factor 1, less specific in the endometrium than in the ovary), and α-methylacyl-coenzyme-A racemase (AMACR, still not validated) [2–5].

Compared to endometrioid and serous carcinoma, little is known about precursors of CC-EC. To date, only one series of putative precursors of CC-EC has been published [6]. In addition, very few cases of CC-EC limited to the endometrial epithelium have been described [7–9]; such cases have been referred to as “endometrial intraepithelial carcinoma (EIC), clear cell-type” or “clear cell EIC” (CC-EIC).

Herein, we report two cases of CC-EIC accompanied by putative CC-EC precursors, discussing the morphological and immunophenotypical aspects and the diagnostic issues related to such lesions.

## Case presentation

### Case 1

**Fig. 1:**
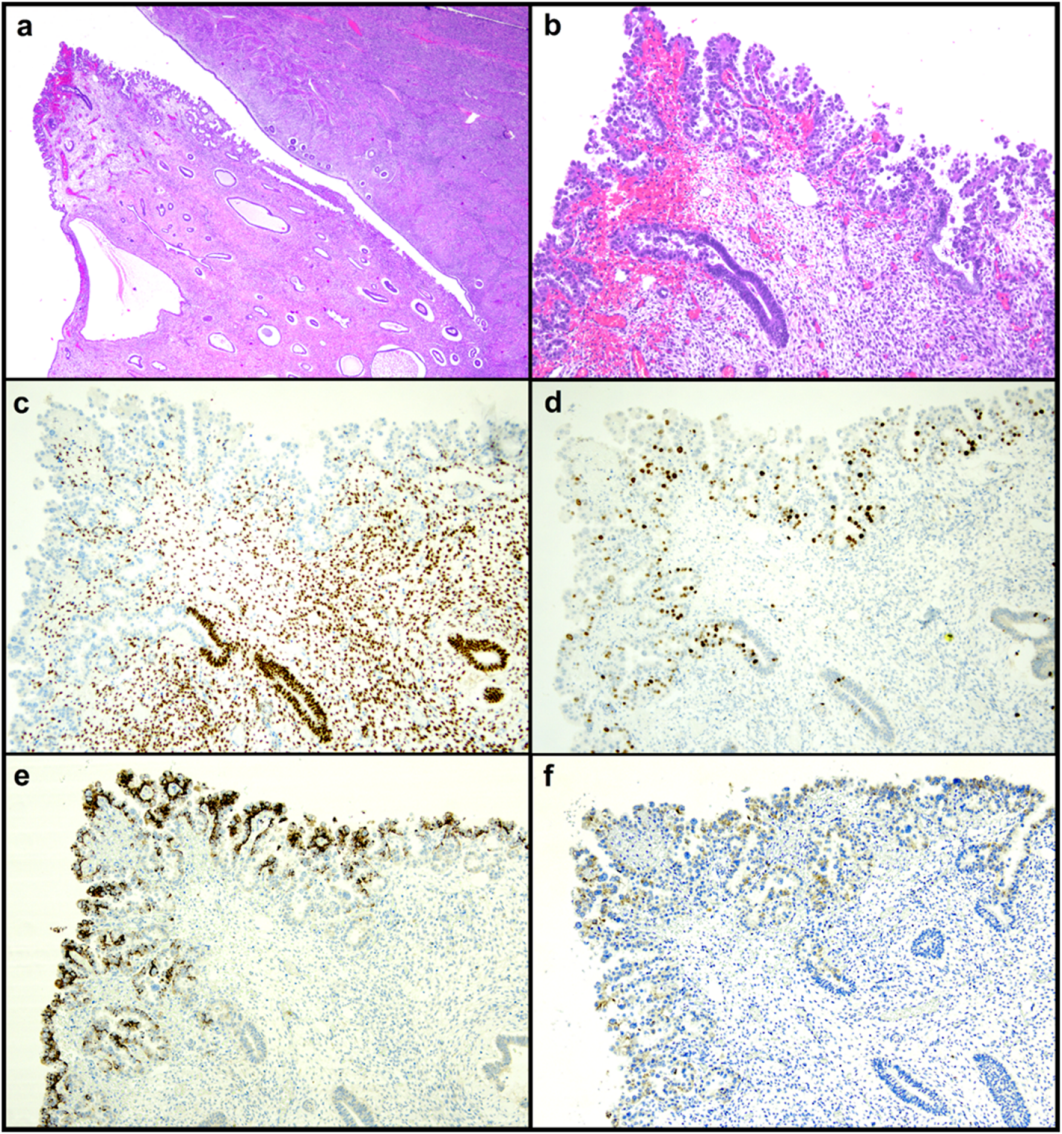
Case 1. Clear cell endometrial intraepithelial carcinoma on the surface of an endometrial polyp. Hematoxylin-eosin at 40X (**a**) and 200X (**b**) magnification. Immunohistochemical stain for estrogen receptor (**c**), Ki67 (**d**), Napsin A (**e**) and AMACR (**f**) (magnification 200X)

A 69-year-old patient underwent hysterectomy due to postmenopausal bleeding; the patient did not refer hormone therapy. On gross examination of the hysterectomy specimen, the endometrial cavity was occupied by a large atrophic-cystic polyp. On histological examination, the surface of the polyp showed a papillary proliferation of atypical cells with large nuclei with dispersed chromatin and evident nucleoli, clear to eosinophilic cytoplasm and hobnail appearance; mitotic activity was noted in the form of occasional mitotic figures. On immunohistochemistry, such proliferation was negative for ER, PR and vimentin (in contrast to the background endometrium), with diffuse p16 expression (variable intensity), increased Ki67 expression, diffuse positivity for Napsin A and focal positivity for AMACR (Fig. 1). Mismatch repair expression was retained. P53 immunostaining showed a null-type mutant pattern; direct sequencing confirmed the presence of a pathogenetic mutation in the exon 4 of *TP53*, i.e. c.487T>A, p.(Tyr163Asn). The subsequent sampling of the entire endometrial cavity showed atrophic endometrium with no further evident lesions. On the account of these findings, a diagnosis of CC-EIC was made. On the endometrial polyp surface and adjacently to CC-EIC, there was a proliferation of atypical cells that shared cytological and immunophenotypical features with CC-EIC (including the aberrant p53 pattern) but lacked the papillary architecture and the expression of Napsin A and AMACR; these features suggested that such lesion might represent a high-grade precancerous lesion of CC-EC (Table 1). Just beneath this lesion, there was a gland lined by eosinophilic, mildly-to-moderately atypical cells with cilia, resembling atypical tubal metaplasia [10]; such area shared with CC-EIC the loss of ER, PR and vimentin, and the diffuse p16 expression (although less intense), which stood out from the surrounding resting endometrium; the expression of Ki67 was higher than in the resting endometrium but definitely lower than in CC-EIC; Napsin A and p450S were negative and p53 immunostaining was wild-type (Table 1). The hypothesis of such area being a precursor of CC-EIC rather than a metaplastic change could not be demonstrated.

**Table 1:** Morphological and immunohistochemical features of normal endometrium and putative CCC precursors

	Resting endometrium	Putative low-grade precursors (metaplasia-like)	Putative low-grade precursors (AEH/EIN-like)	Putative high-grade precursor	CC-EIC	Invasive CC-EC
**nuclear atypia**	none	low-grade	low-grade	high-grade	high-grade	high-grade
**architecture**	flat	flat	flat	flat	papillary	solid
**ER, PR**	positive	negative	positive	negative	negative	negative
**ki67**	low	moderate (focally high)	moderate	high	high	high
**vimentin**	positive	negative	positive	negative	negative	negative
**p16**	weak and patchy	weak/moderate and diffuse	patchy	moderate/strong and diffuse	moderate/strong and diffuse	moderate/strong and diffuse
**p53**	wild-type	wild-type	wild-type	mutant-type	mutant-type	mutant-type
**Napsin A, AMACR**	negative	negative	negative	negative	positive	positive

*AEH/EIN *Atypical Endometrial Hyperplasia/Endometrioid Intraepithelial Neoplasia*, CC-EIC *Clear Cell-Endometrial Intraepithelial Carcinoma*, CCEC *Clear Cell Endometrial Carcinoma

### Case 2

**Fig. 2:**
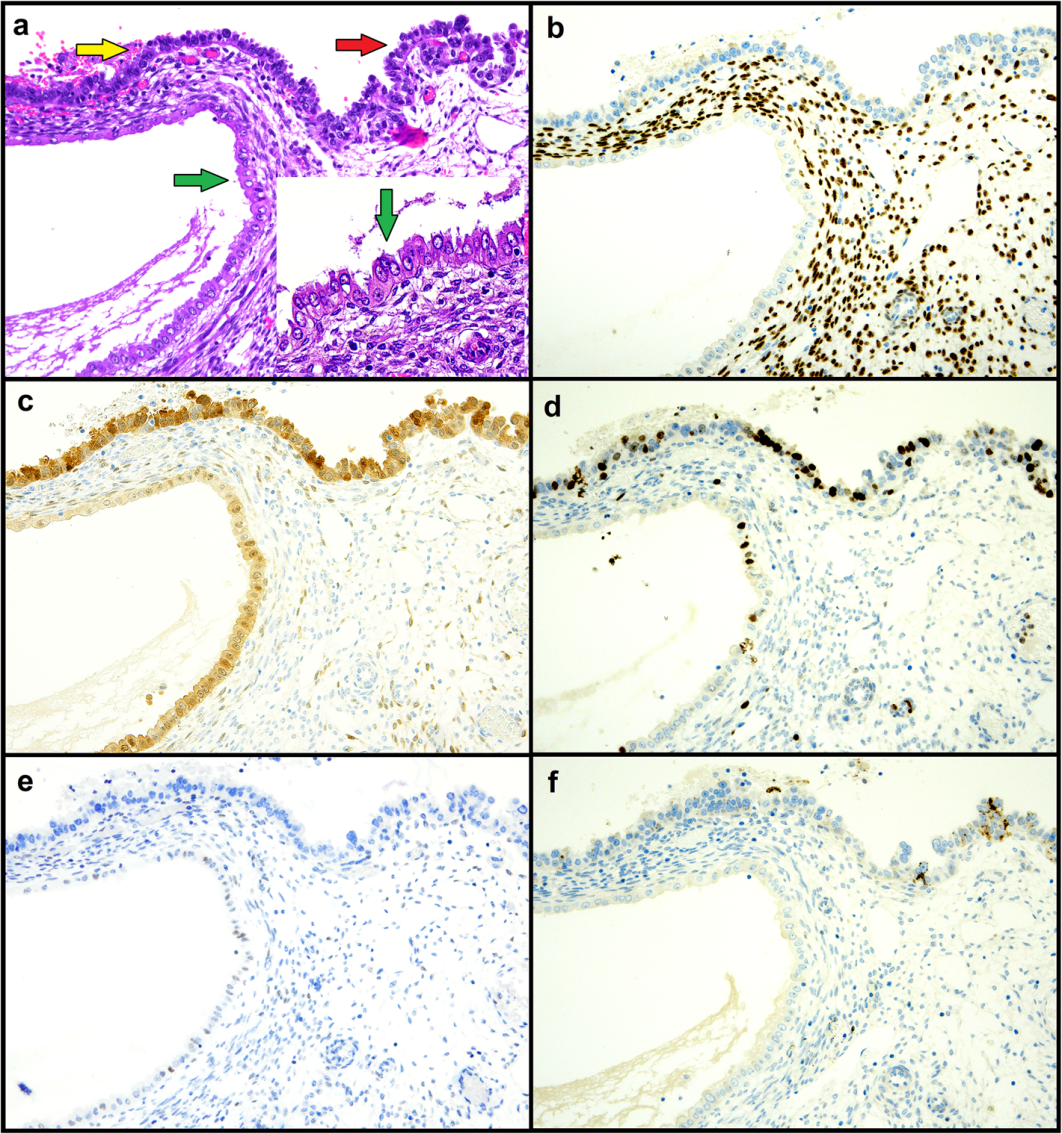
Case 1. Metaplastic-like putative low-grade precursor (green arrow), putative high-grade precursor (yellow arrow) and CC-EIC (red arrow) (magnification 200X). Hematoxylin-eosin with detail of the putative low-grade lesion (**a**) and immunohistochemistry for estrogen (**b**), p16 (**c**), Ki67 (**d**), p53 (**e**) and Napsin A (**f**)

**Fig. 3:**
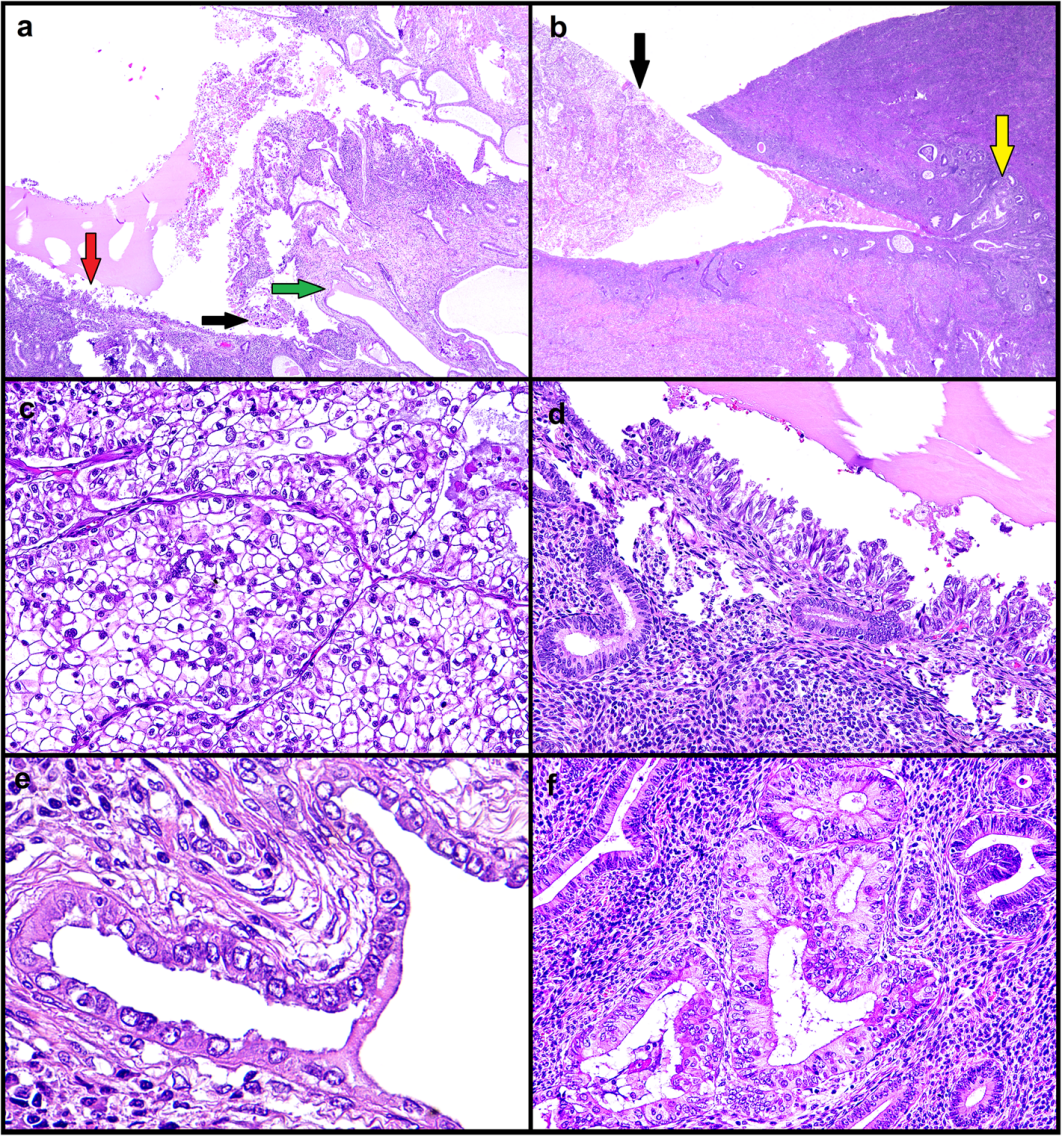
Case 2. Hematoxylin-eosin findings. Panoramic view (**a** magnification 40X) (**b** magnification 20X) of invasive clear cell carcinoma (black arrow) and metaplastic-like putative precursor (green arrow) in en endometrial polyp, with CC-EIC (red arrow) and AEH/EIN-like putative precursor (yellow arrow) in the surrounding endometrium. Details of invasive clear cell carcinoma (**c** magnification 200X), CC-EIC (**d** magnification 200X), metaplastic-like putative low-grade precursor (**e** magnification 400X), AEH/EIN-like putative low-grade precursor (**f** magnification 200X)

**Fig. 4:**
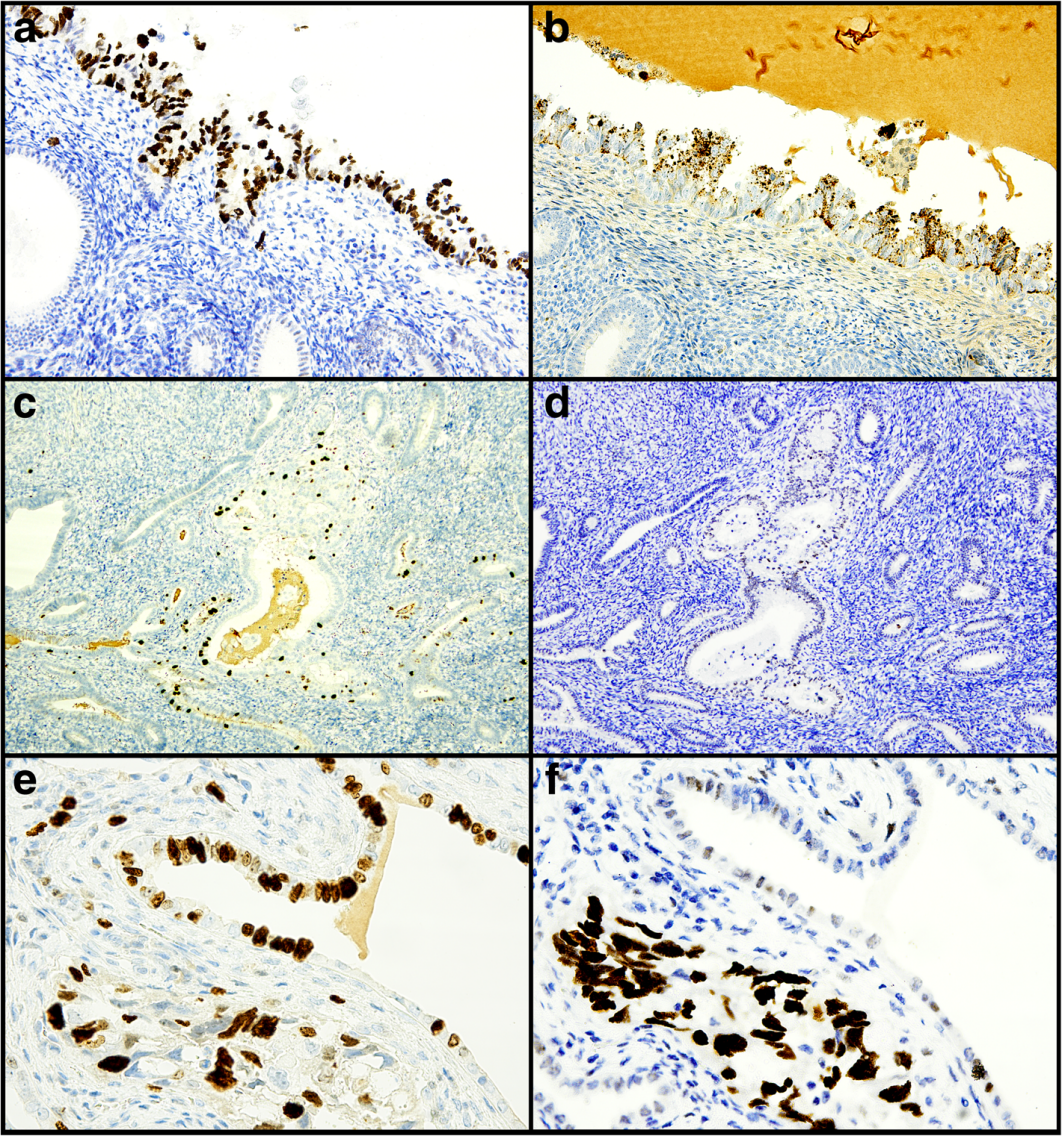
Case 2. P53 overexpression (**a**) and Napsin A positivity (**b**) in CC-EIC (magnification 200X). Increased Ki67 expression (**c**) and p53 wild-type expression (**d**) in AEH/EIN-like putative precursor (magnification 100X). High Ki67 expression (**e**) and p53 wild-type expression (**f**) in metaplastic-like putative precursor (top right) in comparison to invasive clear cell carcinoma (bottom left) (magnification 200X)

A 69-year-old woman with no history of hormone therapy underwent hysterectomy due to a diagnosis of CC-EC on endometrial biopsy; the lesion appeared to arise on a polyp. On the hysterectomy specimen, the invasive CC-EC was limited to the polyp; the endometrium was entirely sampled. Near to the polyp base, an area of CC-EC limited to the endometrial surface lining was observed (Fig. 3). Both the invasive and intraepithelial CC-EC showed p53 overexpression and retained mismatch repair expression, with negativity for ER, PR and vimentin and positivity for Napsin A expression (AMACR was negative instead) (Fig. 4). At the polyp base there was an area of tubal metaplasia with eosinophilic change and nuclear atypia, similar to that described in case 1, with also a similar immunphenotype (but with higher Ki67 expression) (Figs. 1 and 2). Furthermore, in the adjacent endometrium a focal area of complex, crowded glands with altered cytology (reminiscent of atypical endometrial hyperplasia/endometrioid intraepithelial neoplasia, AEH/EIN) and with clear-to-eosinophilic cytoplasm was observed; such area showed increased Ki67 expression compared to the background endometrium, with a p53 wild-type expression, negativity for Napsin A and AMACR (Figs. 3 and 4) and retained ER, PR and vimentin expression (consistent with a more “endometrioid” phenotype) (Table 1). Despite the seemingly “premalignant” appearance and the clear cell changes, the immunophenotype could not support a relationship with CC-EC.

## Discussion

In the last decades, great advances have been achieved in the study of precursor lesions of endometrial carcinoma. For endometrioid carcinoma, AEH/EIN has emerged as a recognized precursor, which is distinct from benign endometrial hyperplasia by morphological, immunophenotypical and molecular features [1].

For serous carcinoma, an intraepithelial form termed “EIC” has been described [1]. Serous EIC (S-EIC) reportedly arises in atrophic endometrium, especially in polyps, and recapitulates the morphological and immunophenotypical features of invasive serous carcinoma (i.e. high-grade nuclear atypia, scalloped profile, cellular tufting, high mitotic index, p53 mutant-type pattern, high Ki67 expression), which allow its identification [1, 4]. Although limited to the endometrial epithelium, the 5th WHO (World Health Organization) classification of Female Genital Tumors recommends to consider S-EIC as a potentially metastatic disease, as it can metastasize to extrauterine sites [11]. On this account, it appears appropriate to consider S-EIC as a small or early serous carcinoma rather than an *in situ* or precancerous lesion.

In addition to S-EIC, an earlier premalignant form termed “endometrial glandular dysplasia” (EmGD) has been proposed [1]. EmGD is described as an intraepithelial lesion arising in atrophic endometrium, characterized by evident nuclear atypia (that is milder than that of S-EIC), increased Ki67 expression and a p53 mutant-type pattern [1, 4, 12, 13]. Remarkably, the p53 mutant pattern is also identifiable in so-called “p53 signature”, which lacks cytologic atypia and is regarded as a very early precursor of serous carcinoma [1, 4, 12]. However, EmGD is not recognized in the current WHO classification of Female Genital Tumors [11].

Both EIC and EmGD have also been proposed as steps of CC-EC carcinogenesis under the name of clear cell type EIC and EmGD (“CC-EIC and CC-EmGD”); however, very few data have been published about these entities [7–9, 13], and the current WHO classification does not list any putative precursor of CC-EC [11].

Based on our and previous reports, CC-EIC appears analogous to S-EIC, as it recapitulates the morphologic and immunophenotypical features of its invasive counterpart [7–9, 13]. On this account, CC-EIC may be defined by the presence of atypical cuboidal, polygonal or hobnail cells with clear-to-eosinophilic cytoplasm, which replace the epithelial lining of endometrial surface and glands with no evidence of invasive disease. In our cases, immunohistochemistry also showed consistent results, with negativity for ER and PR and positivity for Napsin A (in both cases) and AMACR (in Case 1); the presence of p53 abnormalities supported its neoplastic nature, and its relationship with CC-EC in Case 2. Based on these features, CC-EIC would fulfill at least two of the five National Cancer Institute (NCI) requirements to be defined as a precancer [14], since it can be distinguished from both normal endometrium (requirement 3) and CC-EC (requirement 4).

The distinction from reactive/metaplastic changes (requirement 5) may be more difficult, since these may show eosinophilic, hobnail or clear cell morphology and nuclear atypia [11, 15–18]. According to our experience and previously published studies, such changes may show reduced ER, PR and vimentin expression and increased p53, p16 and Ki67 expression [15–18]. Tubal metaplasia may also show nuclear atypia which may raise the concern of EIC [10]. This may result in overtreatment with unnecessary hysterectomy. In S-EIC, the striking features such as pleomorphic nuclei, high mitotic index, mutant-type p53 pattern and high Ki67 expression may exclude benign changes [1, 4, 10]. In CC-EIC, nuclear pleomorphism and mitotic activity are typically milder than serous carcinoma [1], Ki67 may be lower [19], and most CC-ECs lack *TP53* mutations [1]; however, the combination of the typical morphological/immunohistochemical features, as seen in our cases, would still allow a correct diagnosis.

In order to fully recognize CC-EIC as a precursor of CC-EC, it should be proven that CC-EIC is associated with increased risk of CC-EC (requirement 1), and that CC-EC arises from cell within CC-EIC (requirement 2). Although the unavailability of follow-up data and the rarity of early diagnosed CC-EC prevent the assessment of these aspects, the relationship between CC-EIC and CC-EC appears supported by their striking similarity; furthermore, despite being very rare as isolated finding CC-EIC has been reported in association with invasive CC-EC [20].

Whether CC-EIC has metastatic potential like S-EIC remains to be defined. This would impact the patient management, since a potentially metastatic lesion should be treated as an early or small CC-EC. Even in the absence of metastatic potential, the finding of CC-EIC in hysteroscopic specimens (such as atrophic polyps) would still require hysterectomy. Being aware of the possibility of CC-EIC may aid pathologists in not missing such lesion. On this account, if the pathologist is dealing with a neoplasm reminiscent of S-EIC but with inconsistent p53 and Ki67 expression, features of CC-EIC (including the typical immunohistochemical profile) should also be assessed before making a diagnosis of reactive change.

Diagnostic issues are still more complex for earlier precursors of CC-EC, i.e. premalignant intraepithelial lesions termed CC-EmGD by Fadare et al. [13]. Fadare et al. described these putatuive precursors as highly heterogeneous lesions, including isolated glands or surface epithelium displaying cytoplasmic clearing/eosinophilia, hobnail changes, varying degrees of nuclear atypia and increased p53 and Ki67 expression compared to normal endometrium [7]. However, these features may also be found in reactive/metaplastic changes (as discussed above), and these putative precursors were identified as such because they were adjacent to CC-EC. In our cases, three types of putative precursors were identified. In case 1, a putative high-grade precursor was recognized as such based on the p53 mutant-type pattern (identical to that of the adjacent CC-EIC), although it lacked the expression of the typical CC-EC markers such as Napsin A and AMACR. In both case 1 and case 2, areas of putative low-grade precursors with features of atypical tubal metaplasia was identified adjacently to CC-EIC/CC-EC. These areas showed cytoplasmic eosinophilia, nuclear atypia, loss of ER, PR and vimentin and increased Ki67, p16 and p53 expression; none of these features allowed excluding a benign reactive/metaplastic change. In case 2, another putative low-grade precursor showed morphological features reminiscent of AEH/EIN but with cytoplasmic clearing and eosinophilia; immunohistochemistry showed an “endometrioid” phenotype with no similarity to CC-EC. In summary, we were able to recognize a probable CC-EC precursor only in the presence of p53 mutation. Since most CC-ECs have neither p53 mutation nor other known molecular hallmarks, the recognition of their precursors appears nearly impossible. At the time, these lesions cannot met the NCI requirements for precancer. A better definition of the molecular mechanisms that underlie the development of CC-EC appears necessary to define and recognize its precursors.

## Conclusions

In conclusion, our cases, alongside with the previous literature, support that CC-EIC is a real entity which may be diagnosed based on a combination of morphological and immunophenotypical features that are typical of CC-EC, but in the absence of invasive disease. The clinical significance of CC-EIC is undefined, although it is reasonable to assume that it should be managed by hysterectomy. Putative earlier precursors of CC-EC have been described, but with no univocal morphologic or immunophenotypical features that allow their distinction from metaplastic/reactive changes. We hope further studies may shed light on the carcinogenetic pathways and precursors of CC-EC.

## Data Availability

The datasets used and/or analysed during the current study are available from the corresponding author on reasonable request.

## Abbreviations

CC-EC: Clear cell endometrial carcinoma
CC-EIC: Clear cell - endometrial intraepithelial carcinoma
ER: Estrogen receptor
PR: Progesterone receptor
HNF1: Hepatocyte nuclear factor 1
AMACR: Alpha-methylacyl-CoA racemase
EIC: Endometrial intraepithelial carcinoma
AEH/EIN: Atypical endometrial hyperplasia/endometrioid intraepithelial neoplasia
S-EIC: Serous endometrial intraepithelial carcinoma
WHO: World Health Organization
EmGD: Endometrial glandular dysplasia
CC-EIC: Clear cell type endometrial intraepithelial carcinoma
CC-EmGD: Clear cell type endometrial glandular dysplasia
NCI: National Cancer Institute
